# Parallelism Between Sentence Structure and Nominal Phrases in Japanese: Evidence from Scrambled Instrumental and Locative Adverbial Phrases

**DOI:** 10.1007/s10936-022-09843-1

**Published:** 2022-04-06

**Authors:** Katsuo Tamaoka, Takane Ito, Michael P. Mansbridge

**Affiliations:** 1 School of Foreign Languages, Hunan University, Lushan Road (S), Yuelu District, Changsha City, Hunan Province 410082 China; 2 Graduate School of Humanities, Nagoya University, Furo-cho, Chikusaku, Nagoya, Aichi 464-8601 Japan; 3 Graduate School of Interdisciplinary Information Studies, the University of Tokyo, 3-8-1, Komaba, Meguroku, Tokyo, 153-8902 Japan; 4 Research Faculty of Media and Communication, Hokkaido University, Nishi 8-chome, Kita 17-jo, Kitaku, Sappro, Hokaido 060-0817 Japan

**Keywords:** Nominalization, Parallelism, Locative adverbial phrases, Instrumental adverbial phrases, Canonical order, Scrambling

## Abstract

The present study investigated the canonical position of instrumental and locative adverbial phrases in both Japanese sentences and noun phrases to determine whether the canonical positions are parallel. A series of sentence/phrase decision tasks were used to compare sentences with different word-orders, including sentences with S*Adv*OV (S is subject phrase, *Adv* adverb, O object phrase and V verb), *Adv*SOV, S*Adv*OV and SO*Adv*V word orders. S*Adv*OV word order was found to be the most quickly processed, for both instrumental adverbial (Experiment 1) and locative adverbial phrases (Experiment 2). Thus, the canonical position for these adverbial phrases is identified as the position immediately preceding the object (Theme argument). This finding was replicated when the same experimental methods were applied to event-denoting noun phrases. Adverbial adjuncts in the initial position (*Adv*ON, N is noun phrase) were processed more quickly and accurately than noun phrases with adverbial phrases in the second position (O*Adv*N), for both instrumental adverbial (Experiment 3) and locative adverbial phrases (Experiment 4). Therefore, the position immediately preceding the object is the canonical position for both instrumental and locative adverbial phrases in sentences and in noun phrases. In conclusion, this indicates that the base structure of a sentence is shared by its related noun phrase.

## Introduction

Some sentences have corresponding noun phrases with no change of fundamental meaning. Taking an example from Chomsky (1970), the active sentence *the enemy destroyed the city* corresponds to the noun phrase *the enemy’s destruction of the city*. Here, the subject of the noun phrase is marked by the possessive –*s* and the object of the noun phrase is introduced with the preposition *of*. This is referred to as ‘nominalization’. Although the past tense is lost in the noun phrase, both the sentence and the noun phrase carry essentially the same meaning and have similar elements. In this example, the noun *destruction* is a nominal derived from the verb *destroy* and acts as the head of the whole noun phrase. Using the genitive case marker –*no*, nominalization in Japanese is easily accomplished with relatively free word order. Thus, the word order in Japanese, where scrambling is allowed in both sentences and noun phrases, is much more flexible than in English. A study using eye-tracking (Tamaoka & Mansbridge, 2019; Tamaoka et al., 2014) showed longer eye-fixation times for the nominative-marked (-*ga*) noun phrase in scrambled sentences of OS*gap*V (*gap* refers to the original object position) than in its counterpart of the accusative-marked (-*o*) noun phrase in canonical sentences of SOV. Assuming faster processing times for canonically ordered sentences and possibly noun phrases compared to scrambled orders, the present study investigated whether the derived noun phrase (i.e., nominalization) has fundamentally the same syntactic structure as its corresponding sentence. More specifically, the present study addressed this question from two perspectives; (1) by attempting to determine the canonical position of instrumental or locative adverbial phrases in both sentences and noun phrases, and when this was clarified, (2) by determining whether the canonical order of a sentence with an instrumental or a locative adverbial phrase is the same as the canonical order of its corresponding noun phrase.

### A Japanese Sentence and Its Corresponding Noun Phrase and Scrambling

Japanese nominals that can have sentential correlates are formed by adding the genitive case-marker –*no* to the postpositional phrase (PP) and noun phrase (NP) within the whole NP. For instance, a sentence *Gakusei-ga yuubin-de zyuken-o moosikon-da*, N(student)-NOM(-*ga*) N(post)-P(-*de*) N(exam)-ACC(-*o*) V(apply.for)-PAST, ‘The student applied for the exam by post’ has a corresponding noun phrase *Gakusei-no yuubin-de-no zyuken-no moosikomi*, N(student)-GEN(-*no*) N(post)-P(-*de*)-GEN(-*no*) N(exam)-GEN(-*no*) N(application), ‘the student’s application for the exam by post’. In this noun phrase, the nouns *gakusei* ‘the student’ and *zyuken* ‘exam’, and the PP consisting of *yuubin* ‘post’ and the instrumental postposition –*de* are marked with the genitive case marker –*no*. The nominative marker –*ga* and accusative case marker –*o* are obligatorily dropped when the genitive case marker –*no* is added.

Furthermore, Japanese allows flexible word orders in both sentences and noun phrases. In other words, the aforementioned example sentence can be scrambled to *yuubin-de gakusei-ga zyuken-o moosikon-da* by moving [_PP_ N(post)-P(-*de*)] to a higher position than [_NP_ N(student)-NOM(-*ga*)]. This sentence has the corresponding noun phrase *yuubin-de-no gakusei-no zyuken-no moosikomi*. Like the sentence, this noun phrase allows the order of *gakusei-no* [_NP_ N(student)-GEN(-*no*)] and *yuubin-de-no* [_PP_ N(post)-INS(-*de*)-GEN(-*no*)] to be shifted, but both those constituents must be marked with the genitive case marker -*no*. The meaning of the scrambled sentence and its corresponding noun phrase remains the same.

In psycholinguistic experimental studies on scrambling (e.g., Imamura et al., 2016; Koizumi & Tamaoka, 2004, 2006, 2010; Mazuka et al., 2002; Miyamoto & Takahashi, 2004; Tamaoka et al., 2005; Tamaoka et al., 2014; Tamaoka & Mansbridge, 2019; Ueno & Kluender, 2003; Witzel & Witzel, 2016), the canonical order of SOV (S is subject phrase, O object phrase and V verb) was found to be faster to process than the scrambled (i.e., different) order of OSV. The delay in processing time between scrambled OSV-ordered sentences and their SOV canonical counterparts is known as *the scrambling effect*. The sentence processing model of *gap-filling parsing* (Frazier & Clifton, 1989; Frazier & Flores D’Arcais, 1989; Frazier, 1987; Stowe, 1986) provides one possible explanation for the delay with the scrambled OSV order. This scrambling can be explained as a syntactic operation of phrasal movement from the original locus of the object (NP-*o*) in the canonical position to the sentence initial position as in [_CP_ NP-*o*_1_ [_IP_ NP-*ga* [_I’_ [_VP_ *gap*_1_ V] I]]] where IP refers to inflectional phrase and CP to complement phrase. The *gap*_1_ indicates the original position in the canonical order from which the NP*-o*_1_ was moved to the sentence initial position. To process the scrambled sentence, native Japanese speakers must recognize the initial NP-*o* as the filler, and then find its original position in the specifier of VP (*gap*_1_) to establish the filler-gap dependency. Here, due to the degree of syntactic complexity, a canonical SOV-ordered sentence is expected to be processed more quickly than its OSV-ordered scrambled counterpart.

Some of the difficulties thought to be associated with scrambling can be explained in part by limitations in working memory or computational resources (Gibson, 1998), the increased syntactic complexity of the scrambling operation (Hawkins, 2004), expectation of constituents (Levy, 2008) and discourse contexts (Kaiser & Trueswell, 2004; Yano & Koizumi, 2018). Discourse and frequency effects have been found to modulate the overall scrambling effect such that they can attenuate the processing difficulty (Imamura et al., 2016). The eye-tracking studies (Tamaoka & Mansbridge, 2019; Tamaoka et al., 2014) clearly indicated longer eye fixation times in the area (noun phrase) assumed to have a *gap* and in the area of a head verb under the scrambled condition. In these eye-tacking studies, there were no discourse contexts presented prior to the scrambled sentences. In such conditions, scrambled sentences seem to result in a heavier cognitive load for processing; scrambling should be the main source of difficulty in processing a single sentence. Using this nature of the scrambling effect, the present study examines the canonical position of instrumental and locative adverbial phrases.

### Possible Canonical Positions of Multiple Adverbs

Adverbs are optional elements of a sentence, categorized as adjuncts that can be placed in various positions. Koizumi (1993) classified Japanese adverbs into three types based on their canonical positions, (1) adverbs in a modal phrase (MP adverbs) such as *saiwainimo* ‘fortunately’, *tabun* ‘perhaps’, (2) adverbs in an inflection phrase (IP adverbs) such as *kinoo* ‘yesterday’, *mukasi* ‘once upon a time’, and (3) adverbs in a verb phrase (VP adverbs) such as *konagonani* ‘into pieces’, *kossori* ‘secretly’. Each type of adverb is argued to have a canonical position—i.e., a position it must occupy before scrambling.

Canonical positions of various types of adverbs are identified as follows (*Adv* refers to adverbs):

- [_MP_ (MP *Adv*) [_IP_ (IP *Adv*) Subject (IP *Adv*) [_VP_ (VP *Adv*) Object (VP *Adv*) Verb] I] M].

The canonical position of the modal phrase adverb (MP *adv*) *saiwainimo* ‘fortunately’ is located within the modal phrase as in *saiwainimo Kenzi-ga han’nin-o tukamae-ta* ‘Fortunately Kenzi caught a criminal’. Temporal adverbs like *kinoo* ‘yesterday’, *kyoo* ‘today’, or *asita* ‘tomorrow’ are classified as the inflectional phrase adverb (IP *adv*), taking their canonical positions before or after the subject. For example, the canonical positions of the time adverb *kinoo* can be placed either before the subject as in *Kinoo Tomoko-ga kami-o kit-ta* ‘Yesterday Tomoko cut her hair’ or after the subject as in *Tomoko-ga kinoo kami-o kit-ta*.

According to Koizumi (1993), both manner and resultative adverbs are classified as verb phrase adverbs (VP *adv*). There are two possible canonical positions within VP adverbs (i.e., canonically neutral), either before the object or after the object (before the verb), or S*Adv*OV and SO*Adv*V. For example, the canonical position of the resultative adverb *konagonani* ‘into pieces’ can be either before the object as in *Kenzi-ga konagonani kabin-o kowasi-ta* ‘Kenzi broke the vase into pieces’ or after the object (before the verb) as in *Kenzi-ga kabin-o konagonani kowasi-ta*. This claim is supported by an experimental sentence-processing study by Koizumi and Tamaoka (2006), whose reading-time measurements revealed that the canonical position of manner and resultative adverbs is either S*Adv*OV or SO*Adv*V. Furthermore, a corpus study by Namba and Tamaoka (2016) counted frequencies of adverb occurrences, indicating that manner adverbs showed near equal distribution for 50.0% of S*Adv*OV and 48.4% of SO*Adv*V positions. There were only 1.7% in *Adv*SOV. As with the psycholinguistic study by Koizumi and Tamaoka (2006), manner adverbs appear to be canonically neutral between those two positions. In sentences with a resultative adverb, the corpus frequency of word order is 80.7% for SO*Adv*V, 18.0% for S*Adv*OV, and 1.3% for *Adv*SOV. Although manner adverbs appear randomly in either the S*Adv*OV position or the SO*Adv*V position, resultative adverbs appear most frequently in the SO*Adv*V position. Thus, manner and resultative adverbs may have different canonical positions within the VP adverbial position.

### Possible Canonical Positions of Instrumental and Locative Adverbial Phrases

The canonical positions of instrumental and locative adverbial phrases have yet to be clearly defined. In fact, Koizumi and Tamaoka (2006) did not investigate instrumental and locative adverbial phrases ending with -*de* (i.e., [_PP_ NP + Postposition(-*de*)]) in their investigation. These can be placed in three different positions in Japanese sentences: in the sentence-initial position as in *Rimokon-de Tomoko-ga terebi-o kesi-ta* ‘Tomoko turned off the television with remote control’, with the order *Adv*SOV; in the post-subject position (S*Adv*OV) as in *Tomoko-ga rimokon-de terebi-o kesi-ta*; or in the post-object position (SO*Adv*V) as in *Tomoko-ga terebi-o rimokon-de kesi-ta*. It is possible, however, to consider the instrumental and locative adverbial PP phrase with –*de* to have one or two basic positions. As the first step of such an investigation, the present study examines where the canonical position of these might be located.

**Fig. 1:**
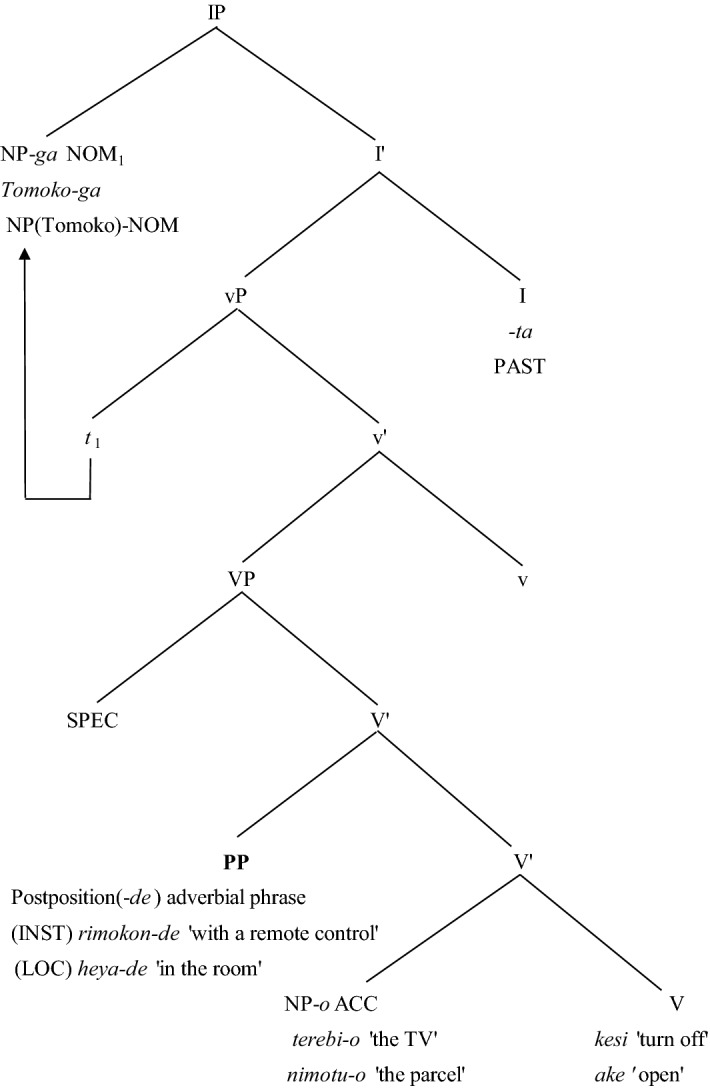
The canonical position of instrumental and locative adverbial phrases at the sentence level *Note*: The PP refers to the assumed canonical position of the adverb. INST refers to instrumental adverbs and LOC to locative adverbs

Because these two types of adverbial phrases, instrumental and locative, are generally taken to modify the whole VP (i.e., the verb and its object without tense) as opposed to IP including tense, it is assumed that they are at the VP level. The potential canonical position (S*Adv*OV) is depicted in Fig. 1. There are two examples in Fig. 1. An example of the instrumental adverbial phrase *remokon-de* ‘by remote control’ is *Tomoko-ga rimokon-de terebi-o kesi-ta* ‘Tomoko turned off the television with remote control’. In this sentence, the adverbial phrase is positioned in the specifier of V’ as the postposition (PP) adverbial phrase. Likewise, the locative adverb *heya-de* ‘in the room’ is also found in the same position as *Tomoko-ga heya-de mimotu-o ake-ta* ‘Tomoko opened the package in the room’.

This S*Adv*OV canonical order is proposed by Vinka (2009) who claims locatives and instrumentals in Japanese are similar to the extent that they both take VPs as arguments. Takezawa (2000; as cited by Ogawa & Niinuma, 2012) claims that locatives and resultatives in Japanese are likely to differ in their ordering in relation to a theme determiner phrase (Theme DP) or the object phrase (O), where the former is likely to precede the Theme (S*Adv*OV), and the latter to follow it (SO*Adv*V) at the VP level (VP adverbs). Prior to the present experiments, it was tentatively assumed that S*Adv*OV is the canonical order for both instrumental and locative adverbial phrases. Alternatively, the order SO*Adv*V is another possibility for the canonical order. However, we hypothesize that S*Adv*OV is canonical, and that the order SO*Adv*V is derived by scrambling.

**Fig. 2:**
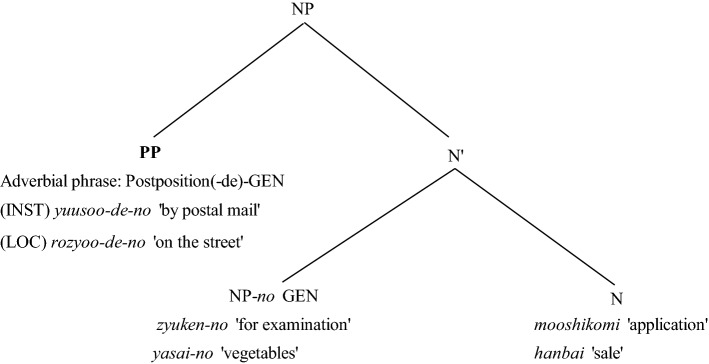
The canonical position of instrumental and locative adverbial phrases at the noun phrase level *Note*: The PP refers to the assumed canonical position of the adverb. INST refers to instrumental adverbs and LOC to locative adverbs

In both examples in Fig. 2, the final nouns of *moosikomi* ‘application’ and *hanbai* ‘sale’ can be considered to have a verb-like argument structure (i.e., have a subject and object), as in their verb forms of *moosikomu* ‘to apply’ and *hanbaisuru* ‘to sell’. Although these noun phrases including instrumental and locative adverbial phrases do not have a subject, they can be considered equivalent to the (S)*Adv*OV-ordered sentence shown in Fig. 1. Thus, it is assumed that the noun phrase with adverb-initial position *Adv*ON (N is noun phrase) would be the canonical order. The same holds for the attested word-order within noun phrases whose heads have an argument structure. Within the noun phrase in Fig. 2, the instrumental and locative adverbial phrases can be re-ordered as O*Adv*V as *zyuken-no yuusoo-de-no moosikomi* and *yasai-no rozyoo-de-no hanbai*. In this study, we hypothesize that *Adv*ON is canonical, and that the order O*Adv*V is derived by scrambling.

It is also assumed that both instrumental and locative adverbial phrases in noun phrases (PP + the genitive maker -*no*) are placed in the initial position *Adv*ON as depicted in Fig. 2. The instrumental adverbial phrase *yuusoo-de-no* ‘by postal mail’ is located at the specifier of NP or in the beginning of the noun phrase in the *Adv*ON order as *Yuusoo-de-no zyuken-no moosikomi* ‘applying for examination by postal mail’. Likewise, the locative adverbial phrase *rozyoo-de-no* ‘on the street’ is also located at the same position of NP specifier as in *Rozyoo-de-no yasai-no hanbai* ‘the sale of vegetables on the street’.

Nominalization is a prevalent cross-linguistic feature, and it is heavily featured in Japanese as well (see Kishimoto, 2006; Miyagawa, 2012; Sugioka, 1992). The nominalized structure may impose the same restrictions on the ordering of elements as a sentential vP structure. However, it is not yet known whether order (i.e., syntactic position) restrictions will also be prevalent within the hierarchal structure of an NP or DP in Japanese.

If native Japanese speakers process the noun phrase in the same manner as they process its corresponding sentence, the scrambling effect should be observed in the processing of an OSV-type of noun phrase as well. This would suggest that the psycholinguistic nature of sentences is shared by their corresponding noun phrases. Instrumental and locative adverbial phrases are excellent candidates for investigating the parallelism of sentences and their noun phrase counterparts because these adverbial phrases allow us to directly compare the syntactic structures of sentences and noun phrases. Subject (S) and object (O) cannot be scrambled in a noun phrase and replaced by the genitive. For instance, the noun phrase, *Nihonzin-no siboo-no torisugi*, N(Japanese)-GEN N(fat)-GEN N(taking-too-much), ‘Too-much eating of fat by Japanese’ does not make sense once it is scrambled as in *Siboo-no nihonzin-no torisugi, N(fat)-GEN N(Japanese)-GEN N(taking-too-much). Hence, the subject phrases must be omitted in investigating scrambling in NPs. In addition, a sentence with a resultative adverb cannot be nominalized: the sentence *Konagona-ni hakai-si-ta*, Adv(into-pieces) V(destroy)-PAST, ‘(I) destroyed (it) into pieces’ cannot be expressed as an NP *Konagona-e-no hakai, ‘destruction into-pieces’. Likewise, a manner adverb used in a sentence has to change its lexical category, from adverb to adjective, in order to be nominalized: the sentence *Subayaku ootoo-si-ta*, Adv(quickly) V(respond)-PAST, ‘I quickly responded (to it)’ becomes a noun phrase where ‘response’ is modified by the adjective ‘quick’, as *subayai ootoo*, Adj(quick) N(response), ‘a quick response’.

To verify the hypothesis that noun phrases are processed in the same way as sentences, this study investigated the existence of the scrambling effect in both sentences and noun phrases with different instrumental and locative adverb orders. In the present study, we conducted four experiments, each relying on the scrambling effect to identify the canonical word order of the structure containing an adverb. These experiments provide empirical evidence supporting (1) the canonical positions for the instrumental and locative adverbial phrases as S*Adv*OV depicted in Fig. 1 at the sentence level and as *Adv*ON in Fig. 2 in the noun phrase and (2) sentences and noun phrases sharing the parallel basic structure (i.e., canonical order).

## Experiment 1: Sentences with Instrumental Adverbial Phrases

Experiment 1 tested whether instrumental adverbial phrases have a canonical position of S*Adv*OV in sentences with transitive verbs.

### Method

#### Participants

Twenty-four graduate and undergraduate students (19 females and 5 males) at a university in Japan, all native speakers of Japanese, participated in Experiment 1. The participants were from various academic backgrounds, and their ages ranged from 18 years and 2 months to 27 years and 8 months. The average age was 21 years and 4 months with a standard deviation of 2 years and 8 months on the day of testing.

#### Materials

Stimuli consisted of sentences that included instrumental adverbial phrases in one of three possible word orders: *Adv*SOV, S*Adv*OV and SO*Adv*V. These stimuli were divided into two classes – semantically coherent “Yes” stimuli, and semantically anomalous “No” stimuli. To create the “Yes” stimulus sentences, twenty-four sentences with an *Adv*SOV structure, e.g., *Pasokon-de Ziro-ga repooto-o kai-ta* ‘Ziro wrote a report with a personal computer’, had their adverbial phrase positions altered into S*Adv*OV, e.g., *Ziro-ga pasokon-de repooto-o kai-ta*, and SO*Adv*V, e.g., *Ziro-ga repooto-o pasokon-de kai-ta*. The only difference among the three types of sentences was the position of adverbial phrase with –*de*. In the same manner, in the case of the “No” stimuli, 24 sets of semantically infelicitous sentences with *Adv*SOV order, e.g., *Yasai-de Ziro-ga Kazuko-ni soodansi-ta ‘Ziro consulted Kazuko with the vegetables’, had the position of their instrumental adverbial PP phrase changed into S*Adv*OV, e.g., *Ziro-ga yasai-de Kazuko-ni soodansi-ta*, and SO*Adv*V, e.g., *Ziro-ga Kazuko-ni yasai-de soodansi-ta*. Again, the only differences among the three were the positions of the adverbial phrases.

Reading times are likely to become shorter when participants read sentences in sequence containing the same words. In order to prevent this effect of repeated encountering, we counterbalanced the list of sentences with a Latin square design. Three lists of sentences were given to three groups of participants (8 each). Each list consisted of 24 sentences (8 each for *Adv*SOV, S*Adv*OV and SO*Adv*V) for “Yes” responses and 24 sentences (8 each of *Adv*SOV, S*Adv*OV and SO*Adv*V) for “No” responses. In addition, 20 dummy sentences (10 semantically coherent and 10 semantically anomalous) were put in each list as filler sentences, such as *Watasi-no tokuina ryoori-wa kareeraisu da* ‘My favorite thing to cook is curry and rice’. A total of 68 stimuli were used, consisting of 24 semantically coherent, 24 semantically anomalous, and 20 dummy sentences. Experiments 1 and 2 were conducted together in a single experiment.

#### Procedure

The present study utilized a reaction time paradigm that measures the elapsed time between the presentation of a sensory stimulus and the participant’s subsequent behavior. This interval is called reaction (or processing) time. The experiment employed a whole sentence correctness decision task, by showing one sentence at a time on a computer screen. The presentation was controlled by Microsoft’s Visual Basic 6.0 + Microsoft DirectX8 computer program. Stimuli with both semantically coherent and semantically anomalous responses were presented to participants in a random order, in the center of a computer screen 600 ms after the appearance of asterisks ‘********’ indicating an eye-fixation point. Participants were asked to decide whether the sentences were semantically acceptable, by pressing a “Yes” or “No” button. They were also asked to answer as quickly as possible, while maintaining accuracy. Twenty-four practice trials were given to the participants prior to actual testing. Participants were expected to process simple sentences with a high accuracy. Sentences with a scrambled order were expected to require longer processing times than the corresponding ones with canonical order, due to syntactic manipulations.

### Results

There was one extreme “Yes” response and one extreme “No” response in reaction times among sentence correctness decision times (i.e., less than 500 ms or longer than 5000 ms). These two extreme responses were excluded from the reaction time data. Furthermore, only stimulus items with correct responses (i.e., the participants answered “Yes” when the sentence made sense and “No” when it did not) were used in the analyses of reaction times. Before performing the analysis, reaction times outside of 2.5 standard deviations plus and minus the mean reaction time at both the high and low ranges were replaced by boundaries indicated by 2.5 standard deviations from the individual means of participants in each condition. Only one reaction time among correctly-responded “Yes” items fell into this category. The means of “Yes” and “No” reaction times and error rates for sentence correctness decisions are presented in Table 1.

**Table 1:** Reaction times and error rates for sentences with instrumental adverbs in experiment 1

Response type	Position of adverb (Adv)	Reaction time (ms)		Error rates (%)	
		*M*	SD	*M* (%)	SD (%)
"Yes" responses	*Adv* SOV	1807	456	6.25	11.66
	*SAdv* OV	1527	361	7.81	12.12
	SO*Adv*V	1663	479	2.60	6.36
"No" responses	*Adv*SOV	1667	409	16.17	20.21
	S*Adv*OV	1674	436	14.33	17.96
	SO*Adv*V	1671	466	15.17	17.73

A series of one-way analyses of variance (ANOVAs) with repeated measures for three sentence types (*Adv*SOV, S*Adv*OV and SO*Adv*V) of “Yes” responses was conducted on reaction times (milliseconds) and error rates (percent), using participant (*F*_1_) and item (*F*_2_) variabilities. There were significant main effects in both participant analysis [*F*_1_(2, 46) = 8.262, *p* < 0.001] and item analysis [*F*_2_(2, 46) = 6.008, *p* < 0.01] for reaction times, but no main effect for error rates [*F*_1_(2, 46) = 2.120, *p* = 0.132, *ns*; *F*_2_(2, 46) = 1.523, *p* = 0.229, *ns*]. Simple contrast comparisons were conducted among the three *Adv*SOV, S*Adv*OV and SO*Adv*V conditions, indicating that S*Adv*OV was the most quickly recognized word order. As for “No” responses (incorrect sentences), neither reaction times [*F*_1_(2, 46) = 0.007, *p* = 0.993, *ns*; *F*_2_(2, 46) = 0.072, *p* = 0.930, *n.*] nor error rates [*F*_1_(2, 46) = 0.365, *p* = 0.696, *ns*; *F*_2_(2, 46) = 1.181, *p* = 0.322, *ns*] showed significant main effects.

### Discussion

Sentences with canonical word order are predicted to be processed more quickly than sentences with scrambled word orders. Experiment 1 clearly indicated that the sentences in which the instrumental adverbial phrases were placed between the subject phrase and the object phrase were processed the most quickly among the three types of word order. Unlike the canonical position of manner and resultative adverbs being either S*Adv*OV or SO*Adv*V (Koizumi & Tamaoka, 2006), the canonical position of instrumental adverbial phrases was only S*Adv*OV. On the other hand, reaction times and error rates for correctly perceived “No” responses are observed to be null main effects, showing a minute difference in reaction times and a similar trend in error rates. This null effect may be a result of participants having multiple ways to reject these stimuli for “No” responses. For example, a participant may have been able to correctly reject the target sentence after comprehending only the semantically implausible part of the sentence (e.g., an adverbial phrase of ‘consult with vegetable’). Importantly, the “No” responses serve as a reference, and are not a major concern to the main argument for the canonical position of adverbial phrases (hereafter the same holds true for the other three experiments).

## Experiment 2: Sentences with Locative Adverbial Phrases

Experiment 2 tested whether locative adverbial phrases have a canonical position (i.e., S*Adv*OV) in sentences with transitive verbs.

### Method

#### Participants

Experiment 2 was carried out at the same time as Experiment 1, so participants were the same in both experiments.

#### Materials

As in Experiment 1, “Yes” responses consisted of sentences with adverbial phrases in the phrase orders of *Adv*SOV, S*Adv*OV and SO*Adv*V. Twenty-four sentences with an *Adv*SOV structure had their adverbial phrase positions altered into S*Adv*OV and SO*Adv*V. In the same way, for the case of “No” responses, 24 sets of semantically anomalous sentences with *Adv*SOV had the position of their locative adverbial phrase changed into S*Adv*OV and SO*Adv*V. A counterbalance with a Latin square design was used to assign participants to different sentences. Since Experiment 2 was conducted with Experiment 1, the rest of the stimulus conditions were the same as Experiment 1.

#### Procedure

The procedure for Experiment 2 was the same as the procedure for Experiment 1.

### Results

There was only one extreme “Yes” response and no extreme “No” responses in reaction times among sentence correctness decision times (i.e., less than 500 ms or longer than 5000 ms). This item was removed from the reaction time data. In addition, only stimulus items of correct responses were used in the analyses of reaction times. The data trimming process was the same as Experiment 1. No reaction times among correctly-responded “Yes” and “No” items fell outside the range of the mean plus or minus 2.5 standard deviations. The means of “Yes” and “No” reaction times and error rates for sentence correctness decisions are presented in Table 2.

**Table 2:** Reaction times and error rates for sentences with locative adverbs in experiment 2

Response type	Position of adverb (Adv)	Reaction time (ms)		Error rates (%)	
		*M*	SD	*M* (%)	SD (%)
"Yes" responses	*Adv* SOV	1740	440	5.88	8.35
	*SAdv* OV	1626	365	4.83	9.08
	SO*Adv*V	1866	530	7.46	10.51
"No" responses	*Adv*SOV	1719	410	15.17	17.73
	S*Adv*OV	1635	423	15.25	22.91
	SO*Adv*V	1667	434	16.67	20.06

A series of ANOVAs with repeated measures for three sentence types (*Adv*SOV, S*Adv*OV and SO*Adv*V) of “Yes” responses was conducted on reaction times and error rates, using participant (*F*_1_) and item (*F*_2_) variabilities. Significant main effects were found in both participant analysis [*F*_1_(2, 46) = 9.255, *p* < 0.001] and item analysis [*F*_2_(2, 46) = 6.337, *p* < 0.01] for reaction times, but no main effect for error rates [*F*_*1*_(2, 46) = 1.028, *p* = 0.366, *ns*; *F*_*2*_(2, 46) = 0.603, *p* = 0.552, *ns*]. Simple contrast comparisons were conducted among the three conditions, *Adv*SOV, S*Adv*OV and SO*Adv*V, again indicating that S*Adv*OV was the most quickly processed phrase order. As for “No” responses (incorrect sentences), neither reaction times [*F*_1_(2, 46) = 1.007, *p* = 0.373, *ns*; *F*_2_(2, 46) = 0.905, *p* = 0.416, *ns*] nor error rates [*F*_1_(2, 46) = 0.315, *p* = 0.731, *ns*; *F*_2_(2, 46) = 0.063, *p* = 0.940, *ns*] showed significant main effects.

### Discussion

As in Experiment 1, Experiment 2 indicated that sentences with S*Adv*OV phrase order were the most quickly processed among sentences of three different phrase orders. Thus, as with instrumental adverbial phrases, S*Adv*OV must be the canonical position of locative adverbial phrases. Once again, this result differed from the canonical position of manner and resultative adverbial phrases identified as either S*Adv*OV or SO*Adv*V (Koizumi & Tamaoka, 2006).

## Experiment 3: Noun Phrases with Instrumental Adverbial Phrases

Experiment 3 examined whether instrumental adverbial phrases have the canonical position of *Adv*ON in noun phrases.

### Method

#### Participants

Twenty-four graduate and undergraduate students (8 females and 16 males) at a university in Japan, all native speakers of Japanese, participated in Experiment 3. These participants did not participate in Experiments 1 and 2. Ages ranged from 19 years and 2 months to 26 years and 9 months. The average age was 21 years and 3 months with a standard deviation of 1 years and 7 months on the day of testing.

#### Materials

“Yes” responses consisted of noun phrases with instrumental adverbial phrases with the orders *Adv*ON and O*Adv*N. Twenty-four *Adv*ON noun phrases, e.g., *yuubin-de-no zyuken-no moosikomi* ‘application for entrance examination by mail’, were prepared and had their adverbial phrase positions altered into O*Adv*N, e.g., *zyuken-no yuubin-de-no moosikomi*. The only difference between the two types of noun phrases was the position of the instrumental adverbial phrase with -*de*. For the case of “No” responses, a set of the same number of semantically anomalous noun phrases with *Adv*ON, e.g., *densirenzi-de-no obentoo-no kiritori* ‘cutting a lunch box by microwave’, were prepared and the adverbial phrase position shifted into O*Adv*N as in *obentoo-no densirenzi-de-no kiritori*. Again, the only difference between noun phrases was the adverbial phrase position. These noun phrases are semantically unacceptable, so the participants were expected to reject them as incorrect phrases by pressing the "No" key.

In order to prevent the problem of repeatedly encountering noun phrases with the same words, a counterbalanced (or Latin square) design was used to assign participants to different noun phrases. Two lists of noun phrases were given to two groups of participants (12 each). Each list consisted of 24 coherent noun phrases (12 each for *Adv*ON and O*Adv*N) for the “Yes” responses and 24 anomalous noun phrases (12 each for *Adv*ON and O*Adv*N) for the “No” responses. In addition, 20 dummy noun phrases (10 correct and 10 incorrect) were added in each list as filler noun phrases, e.g., *hanagara-no kawaii sukaato* ‘a cute skirt with pictures of flowers’. The total of 68 noun phrases were used for Experiment 3, consisting of 24 semantically coherent, 24 semantically anomalous, and 20 dummy noun phrases. Experiments 3 and 4 were conducted together in a single experimental trial.

#### Procedure

The experiment employed a whole-phrase correctness decision task, by showing one noun phrase at a time on a computer screen. The presentation was controlled by Microsoft’s Visual Basic 6.0 + Microsoft DirectX8 computer program. Stimuli with both semantically coherent and semantically anomalous responses were presented to participants in a random order, in the center of a computer screen 600 ms after the appearance of asterisks ‘********’ indicating an eye-fixation point. Participants were asked to decide whether the noun phrases made sense, by pressing a “Yes” or “No” button. They were additionally asked to answer as quickly as possible, while maintaining accuracy. Twenty-four practice trials were given to the participants prior to actual testing.

### Results

There were two extreme “Yes” responses but no extreme “No” responses in reaction times among noun phrase correctness decision times (i.e., less than 500 ms or longer than 5000 ms). These extreme responses were removed from the reaction time data. In addition, only stimulus items with correct responses were used in the analyses of reaction times. The data trimming process was the same as Experiments 1 and 2. Four reaction times for “Yes” items and 11 reaction times for “No” items fell outside the range of the mean plus or minus 2.5 standard deviations. The means of “Yes” and “No” reaction times and error rates for noun phrase correctness decisions are presented in Table 3.

**Table 3:** Reaction times and error rates for noun phrases with instrumental adverbs in experiment 3

Response type	Position of adverb (Adv)	Reaction time (ms)		Error rates (%)	
		*M*	SD	*M* (%)	SD (%)
"Yes" responses	*Adv* ON	1909	572	8.33	9.83
	O*Adv* N	2263	720	16.67	13.90
"No" responses	*Adv*ON	1937	490	2.08	5.63
	O*Adv*N	2000	632	2.08	5.63

A series of ANOVAs with repeated measures for two noun phrase types (*Adv*ON and O*Adv*N) of “Yes” responses was conducted on reaction times and error rates, using participant (*F*_1_) and item (*F*_2_) variabilities: Two adverbial phrase positions, the adverbial phrase initial position and the adverbial phrase second position, were examined in the processing of noun phrases with instrumental adverbial phrases. The analyses indicated that noun phrases of the adverbial phrase initial position (i.e., *Adv*ON) were processed more quickly [*F*_*1*_(1, 23) = 18.846, *p* < 0.001; *F*_*2*_(1, 23) = 11.158, *p* < 0.01] and more accurately [*F*_*1*_(1, 23) = 5.750, *p* < 0.05; *F*_*2*_(1, 23) = 9.095, *p* < 0.01] than noun phrases with the adverbial phrase second position (i.e., O*Adv*N). For “No” responses (incorrect noun phrases), neither reaction times [*F*_1_(1, 23) = 0.354, *p* = 0.558, *ns*; *F*_2_(1, 23) = 0.186, *p* = 0.671, *ns*] nor error rates [*F*_1_(1, 23) = 0.000, *p* = 1.000, *ns*; *F*_2_(1, 23) = 0.000, *p* = 0.984, *ns*] showed significant main effects.

### Discussion

Experiment 3 indicated that the canonical position of instrumental adverbial phrases in noun phrases is *Adv*ON (adverb initial position). This result replicates the general findings of Experiment 1 for the sentence structure. Thus, this provides evidence that nominal phrases are likely to adhere to similar restrictions of canonical order as the sentence structure.

## Experiment 4: Noun Phrases with Locative Adverbial Phrases

Experiment 4 further investigated whether the canonical position of locative adverbial phrases is also *Adv*ON in noun phrases.

### Method

#### Participants

Experiment 3 was carried out with Experiment 4, so participants were the same in both experiments.

#### Materials

As in Experiment 3, “Yes” responses were noun phrases with locative adverbial phrases in the phrase structure of *Adv*ON and O*Adv*N. Twenty-four semantically coherent noun phrases with *Adv*ON were altered so that their adverbial phrase position was O*Adv*N. The only difference between two types of noun phrases was the position of the locative adverbial phrase with –*de*. In the case of “No” responses, 24 sets of semantically anomalous noun phrases *Adv*ON were prepared with the adverbial phrase position shifted to O*Adv*N. As in Experiment 3, a counterbalanced (or Latin square) design was used to assign participants to different noun phrases. Because Experiment 4 was conducted with Experiment 3, the rest of the stimulus conditions were the same.

#### Procedure

The procedure for Experiment 4 was the same as the procedure for Experiments 1, 2 and 3.

### Results

There were two extreme “Yes” responses and no extreme “No” responses in reaction times among noun phrase correctness decision times (i.e., less than 500 ms or longer than 5000 ms). Once again, these extreme outliers were removed from the reaction time data. Additionally, only stimulus items of correct responses were used in the analyses of reaction times. The data trimming process was the same as Experiments 1, 2 and 3. Two reaction times for “Yes” items and six reaction times for “No” items fell outside the cutoff boundary of the mean plus or minus 2.5 standard deviations. The means of “Yes” and “No” reaction times and error rates for noun phrase correctness decisions are presented in Table 4.

**Table 4:** Reaction times and error rates for noun phrases with locative adverbs in experiment 4

Response type	Position of adverb (Adv)	Reaction time (ms)		Error rates (%)	
		*M*	SD	*M* (%)	SD (%)
"Yes" responses	*Adv* ON	1728	436	6.94	10.90
	O*Adv*N	2031	633	15.28	16.24
"No" responses	*Adv*ON	1764	460	4.17	8.86
	O*Adv*N	1906	470	3.47	6.91

A series of ANOVAs with repeated measures for the two noun phrase types (*Adv*ON and O*Adv*N) of “Yes” responses was conducted on reaction times and error rates. Two adverbial phrase positions, the adverbial phrase initial position and the adverbial phrase second position, were examined in the processing of noun phrases with locative adverbial phrases. The analyses indicated that noun phrases with adverbial phrases in initial position (i.e., *Adv*ON) were processed more quickly [*F*_*1*_(1, 23) = 17.751, *p* < 0.001; *F*_*2*_(1, 23) = 10.456, *p* < 0.01] and more accurately [*F*_*1*_(1, 23) = 4.312, *p* < 0.05; *F*_*2*_(1, 23) = 4.545, *p* < 0.05] than noun phrases with adverbial phrase in second position (i.e., O*Adv*N). As for “No” responses (incorrect noun phrases), reaction times for noun phrases with adverbial phrases in initial position (i.e., *Adv*ON) were shorter than for noun phrases with adverbial phrases in second position (i.e., O*Adv*N) for the participant analysis [*F*_1_(1, 23) = 4.749, *p* < 0.05], but this result did not hold for the item analysis [*F*_2_(1, 23) = 0.977, *p* = 0.333, *ns*]. Thus, we interpret this main effect result as not significant. There was no significant main effect on error rates [*F*_1_(1, 23) = 0.074 *p* = 0.788, *ns*; *F*_2_(1, 23) = 0.097, *p* = 0.758, *ns*].

### Discussion

Experiment 4 indicated that, as with instrumental adverbial phrases in Experiment 3, the adverbial initial position of *Adv*ON was the canonical position of locative adverbial phrases in noun phrases.

## General Discussion

The present study conducted four experiments to investigate whether the base structure of a sentence is shared by its related noun phrase. As depicted in Figs. 1 and 2, the present study hypothesized that instrumental and locative adverbial phrases with –*de* have the same canonical position, S*Adv*OV for sentences, and *Adv*ON for noun phrases. Extending on prior studies of the scrambling effect in Japanese (e.g., Koizumi & Tamaoka, 2004, 2006, 2010; Mazuka et al., 2002; Tamaoka et al., 2005; Tamaoka et al., 2014; Tamaoka & Mansbridge, 2019), which showed that canonical word orders are processed more quickly than scrambled orders, the present study investigated the canonical position of instrumental and locative adverbial phrases.

Experiments 1 and 2 were conducted to find the canonical position of instrumental and locative adverbial phrase marked by –*de* within sentences. Among the three possible phrasal orders of *Adv*SOV, S*Adv*OV and SO*Adv*V, the order of S*Adv*OV was found to be the most quickly processed, both in the case of instrumental in Experiment 1 and locative in Experiment 2. Thus, the canonical position for these adverbial phrases can be identified as the position between the subject and the object. Experiments 3 and 4 examined the canonical position of instrumental and locative adverbial phrases in nominal phrases. The result showed for both the instrumental adverbial phase in Experiment 3 and the locative adverbial phrase in Experiment 4, the adverbial phrase initial position (i.e., *Adv*ON order) was processed more quickly and more accurately than the adverbial phrase second position (i.e., O*Adv*N order). These results suggest that the canonical adverbial phrase position for sentences, S*Adv*OV or PP position in Fig. 1, remains the same in the case of noun phrases, *Adv*ON or PP position in Fig. 2.

Takezawa (2000; as cited by Ogawa & Niinuma, 2012) claims that locatives and resultatives in Japanese are likely to differ in their ordering in relation to a theme determiner phrase (Theme DP) or an object phrase (O), where the former is likely to precede the Theme (S*Adv*OV), and the latter to follow it (SO*Adv*V) at the VP level (VP adverbs). Furthermore, although adverbial phrases are adjuncts and thought to be optional pieces of a sentence, the present study showed that adverbial phrases have canonical base positions in both a sentence and a noun phrase.

According to Koizumi’s (1993; see also Kishimoto, 2006; Miyagawa, 2012; Sugioka, 1992) categorization, the instrumental and locative adverbial phrases tested in this study should be classified as VP adverbs. Under this assumption, their canonical position is defined either S*Adv*OV or SO*Adv*V. In fact, the psycholinguistic study by Koizumi and Tamaoka (2006) indicated that the canonical position of manner and resultative adverbs is as either S*Adv*OV or SO*Adv*V. However, instrumental and locative adverbial phrases with –*de*, those tested in Experiments 1 and 2 for sentences, and Experiments 3 and 4 for noun phrases, turned out to have only one canonical position: S*Adv*OV for a sentence (PP position in Fig. 1), and *Adv*ON for noun phrases (PP position in Fig. 2). Additional support for this order is found in the self-paced reading study by Nambu and Nakatani (2014). Using nominative-genitive alternation, they also provide evidence that locative adverbial phrases (i.e., PPs) are likely to be located under the specifier of VP giving the order S*Adv*OV.

The ultimate purpose of the present experiments was to examine whether the basic word order of a sentence would be shared by its corresponding noun phrases. The four experiments in the present study found that the canonical position for instrumental and locative adverbial phrases remains parallel across both sentences and noun phrases. The adverbial phrase position of S*Adv*OV in sentence is considered equivalent to *Adv*ON for a noun phrase. This study’s findings support the assumption that the base structure of a sentence is shared by its related noun phrase.

## Appendix 1: Sentences used in Experiments 1 and 2

### Semantically Coherent Sentences with Instrumental Adverbial Phrases in Experiment 1

1. 次郎がバットで和子を殴った.

*Ziroo-ga batto-de Kazuko-o nagut-ta.*

N(Ziroo)-NOM *Adv*(bat-de) N(Kazuko)-ACC V(hit)-PAST.

Ziro struck Kazuko with a bat.

2. 太郎がピストルで友子を殺した.

*Taroo-ga pisutoru-de Tomoko-o korosi-ta.*

N(Taroo)-NOM *Adv*(pistol-de) N(Tomoko)-ACC V(kill)-PAST.

Taro killed Tomoko with a gun.

### Semantically Anomalous Sentences with Instrumental Adverbial Phrases in Experiment 1

1. 健二が目薬でセーターを手術した.

*Kenzi-ga megusuri-de seetaa-o syuzyutusi-ta.*

N(Kenzi)-NOM *Adv*(eye-drop-de) N(sweater)-ACC V(operate)-PAST.

Kenzi operated a sweater with an eye-dropper.

2. 和子が糸で高速道路を手伝った.

*Kazuko-ga ito-de koosokudooro-o tetudat-ta.*

N(Kazuko)-NOM *Adv*(string-de) N(a highway)-ACC V(help)-PAST.

Kazuko helped a highway with a string.

### Semantically Coherent Sentences with Locative Adverbial Phrases in Experiment 2

1. 健二がお化け屋敷で和子を驚かした.

*Kenzi-ga obakeyasiki-de Kazuko-o odorokasi-ta.*

N(Kenzi)-NOM *Adv*(a haunted house-de) N(Kazuko)-ACC V(surprise)-PAST.

Kenzi surprised Kazuko in a haunted house.

2. 友子が雑貨屋で花瓶を壊した.

*Tomoko-ga zakkaya-de kabin-o kowasi-ta.*

N(Tomoko)-NOM *Adv*(general store-de) N(flower vase)-ACC V(break)-PAST.

Tomoko broke a flower vase in the general store.

### Semantically Anomalous Sentences with Locative Adverbial Phrases in Experiment 2

1. 友子が警察で太郎を読んだ.

*Tomoko-ga keesatu-de Taroo-o yon-da.*

N(Tomoko)-NOM *Adv*(the police station-de) N(Taro)-ACC V(read)-PAST.

Tomoko read Taro at the police station.

2. 次郎が野球場で手紙を叱った.

*Ziroo-ga yakyuuzyoo-de tegami-o sikat-ta.*

N(Ziroo)-NOM *Adv*(the baseball stadium-de) N(a letter)-ACC V(admonish)-PAST.

Ziro admonished a letter at the baseball stadium.

## Appendix 2: Complex noun phrases used in Experiments 3 and 4.

### Semantically Coherent Noun Phrases with Instrumental Adverbial Phrases in Experiment 3

1. スキャナーでの画像の取り込み.

*sukyanaa-de-no gazoo-no torikomi.*

*Adv*(scanner-*de*)-GEN N(an image)-GEN N(import).

import of an image with a scanner.

2. 郵送での受験の申し込み.

*yuusoo-de-no zyuken-no moosikomi.*

*Adv*(postal mail-*de*)-GEN N(examination)-GEN N(apply).

application for examination by postal mail.

### Semantically Anomalous Noun Phrases with Instrumental Adverbs in Experiment 3

1. 電子レンジでのお弁当の切り取り.

*densirenzi-de-no obentoo-no kiritori.*

*Adv*(microwave-*de*)-GEN N(lunch)-GEN N(cutout).

cutout of lunch with a microwave.

2. 時計での試験時間の横取り.

*tokee-de-no sikenzikan-no yokodori.*

Adv(clock-*de*)-GEN N(examination time)-GEN N(interception).

interception of examination time by clock.

### Semantically Coherent Noun Phrases with Locative Adverbial Phrases in Experiment 4

1. 空港での荷物の持ち運び.

*kuukoo-de-no nimotu-no motihakobi.*

*Adv*(airport-*de*)-GEN N(luggage)-GEN N(transfer).

transfer of luggage at the airport.

2. 球場での監督の胴上げ.

*kyuuzyoo-de-no kantoku-no dooage.*

Adv(baseball stadium-*de*)-GEN N(a coach)-GEN N(victory toss).

A coach’s victory toss at the baseball stadium.

### Semantically Anomalous Noun Phrases with Locative Adverbial Phrases in Experiment 4

1. 砂漠でのたばこの引き延ばし.

*sabaku-de-no tabako-no hikinobasi.*

*Adv*(desert-*de*)-GEN N(a cigarette)-GEN N(stretching).

stretching of a cigarette in the desert.

2. 海岸でのボールの激励.

*kaigan-de-no booru-no gekiree.*

*Adv*(the beach-*de*)-GEN N(a ball)-GEN N(encouragement).

encouraging a ball at the beach.
